# The Art of Reprogramming for Regenerative Medicine

**DOI:** 10.3389/fcell.2022.927555

**Published:** 2022-06-30

**Authors:** Junqi Kuang, Tao Huang, Duanqing Pei

**Affiliations:** ^1^ Institute of Biology, Westlake Institute for Advanced Study, Hangzhou, China; ^2^ Laboratory of Cell Fate Control, School of Life Sciences, Westlake University, Hangzhou, China; ^3^ College of Life Sciences, Zhejiang University, Hangzhou, China

**Keywords:** cell reprogramming, functional cells, cell replacement therapy, chromatin accessibility dynamics, transdifferentiation

## Abstract

Traditional pharmaceuticals in the forms of small chemical compounds or macromolecules such as proteins or RNAs have provided lifesaving solutions to many acute and chronic conditions to date. However, there are still many unmet medical needs, especially those of degenerative nature. The advent of cell-based therapy holds the promise to meet these challenges. In this review, we highlight a relatively new paradigm for generating or regenerating functional cells for replacement therapy against conditions such as type I diabetes, myocardial infarction, neurodegenerative diseases and liver fibrosis. We focus on the latest progresses in cellular reprogramming for generating diverse functional cell types. We will also discuss the mechanisms involved and conclude with likely general principles underlying reprogramming.

## Introduction

Cellular reprogramming refers to a process of cell fate transition, i.e., converting one type of cells to another. The field has a long and rich history starting from the pioneering work of Gurdon in the 1950s. The initial success in frog cloning highlighted the potential of reprogramming fully differentiated nuclei back to totipotent state that can give rise to a new frog (Gurdon, 1962). Three decades later, MyoD was used to convert the mouse embryonic fibroblasts (MEFs) into myoblasts in 1987, marking the first transcription factor or TF to convert cell fate (Davis et al., 1987). In 2006, Takahashi and Yamanaka used 4 TFs, Oct4, Sox2, Klf4 and Myc to convert MEFs to induced pluripotent stem cells (iPSCs) (Takahashi and Yamanaka, 2006; Takahashi et al., 2007). In addition, numerous functional cell types have been reported through reprogramming, including neurons (Vierbuchen et al., 2010; Barker et al., 2018), myoblasts (Davis et al., 1987), cardiomyocytes (Ieda et al., 2010; Qian et al., 2012), hepatocytes (Huang et al., 2011; Sekiya and Suzuki, 2011) and pancreatic β-cells (Zhou et al., 2008). Together, these studies not only demonstrated the feasibility of generating desired cell types from somatic cells, but also provided a rational system to analyze cell fate control.

Despite tremendous advances so far, concerns such as tumorigenicity and efficiency have hampered efforts to implement reprogramming technologies towards human therapies. Alternative approaches such as direct programming *in vivo* or transdifferentiation can provide more appropriate microenvironment with adequate and suitable biochemical and biophysical conditions to make induced mature cells involved into the surrounding tissues and replenish the loss of functional cells in the injured organs (Wang H. et al., 2021). Nevertheless, there are challenges ahead that require more careful investigations, leading to better solutions to issues such as low efficiencies, lack of standard, as well as safe and specific delivery (Fu and Srivastava, 2015; Gascon et al., 2017; Barker et al., 2018; Wang H. et al., 2021). In recent years, the field has achieved substantial progresses in solving these problems. Here we review these novel and promising advances by different cell types and summarize the mechanism and general principles underlying the process of reprogramming for generation for functional cells.

## Skeletal Myogenesis

MYOD was the first TF known to mediate lineage fate conversion, although earlier hints were reported on myoblast generation by treatment with DNA methylation inhibitor 5-Azacytidine that can target the locus of MyoD and activate the expression (Constantinides et al., 1977; Davis et al., 1987). Since then, MYOD has become the most used factor for inducing the reprogramming of skeletal muscle lineage (Chal and Pourquie, 2017). Furthermore, MYOD has been used to induce the direct differentiation of human embryonic stem cells (hESCs) and human-induced pluripotent stem cells (hiPSCs) to myogenic cells, which can be utilized to transplant to alleviate the symptoms of mouse muscle diseases like Duchenne or Miyoshi muscular dystrophies (Goudenege et al., 2012; Saho et al., 2016; Young et al., 2016). The combination of MYOD overexpression and treatment with three chemical molecules CHIR99021, Forskolin and RepSOX could convert mouse fibroblasts into expandable induced PAX7^+^ myogenic progenitor cells (iMPCs) (Bar-Nur et al., 2018). Mechanically, the characteristics of fibroblasts cell fate would be lost gradually, which is prior to the acquirement of stem cell properties during the converting process where Tet regulated DNA demethylation plays a critical role (Yagi et al., 2021).

Interestingly, alternative factors such as transcription factors PAX3/PAX7 have been shown to induce myogenic differentiation in mouse and human pluripotent stem cells (Darabi et al., 2008; Darabi et al., 2012; Quattrocelli et al., 2015; Carrio et al., 2016). Compared with MYOD triggered transdifferentiation, the PAX3/PAX7 approach takes advantage of embryonic body (EB) differentiation followed with sorting by cell surface markers like PDGFRα (CD140a) or CD56 to enrich myogenic cells (Chal and Pourquie, 2017). While the *in vitro* induced myogenic progenitors by conditional expression of Pax3/Pax7 were immature and resemble embryonic/fetal myoblasts, these cells are functionally mature and have long-term regenerative capability upon transplantation (Incitti et al., 2019).

In addition to transcription factors, bioactive nanomaterials have also been utilized to promote the myogenic differentiation. Monodispersed gold and gold-silver nanoparticles (AuNPs and Au-AgNPs) can be used for the attachment and proliferation of myoblasts and facilitate the myogenic differentiation of myoblasts through activating p38α mitogen-activated protein kinase pathway and enhancing the expression of myogenic genes MyoD, MyoG and Tnnt-1 (Ge et al., 2018). Polypyrrole (PPy) is another frequently used biomaterials that has been reported to promote the myogenic differentiation *in vitro* (Li et al., 2019). However, the material has poor degradability and low solubility, limiting its use *in vivo* for regenerative medicine (Li et al., 2019). Recently, Zhou et al. designed an injectable multifunctional polypyrrole@polydopamine (PPy@PDA) crosslinked nanocomposite hydrogel to overcome the drawbacks mentioned above, and they constructed the nanocomposite-crosslinked Pluronic F-127 (F127)-polycitrate matrix (FPCP) to accomplish the enhancement of skeletal muscle repair and regeneration *in vivo* (Zhou et al., 2021). The combination of advanced materials as well as engineered cell types may ultimately provide the solution to myogenic therapies in patients.

### Islet β-cells

The pancreatic islet is a specialized organ with both endocrine (islets of Langerhans) and exocrine (acinar cells and ductal cells) functions (Zhou and Melton, 2018). Exocrine cells, which make up the majority of islets, have strong regenerative capacity whereby the exocrine pancreas could restore rapidly when acute pancreatitis occurs. On the other hand, the endocrine cells, only accounting for the 5% of pancreatic islet, have little capability of regeneration (Zhou and Melton, 2018). Islet β-cells, as one of pancreatic endocrine cell types, can secrete insulin especially and are selectively destroyed in type I diabetes. Replenishing these patients’ pancreatic cells with functional β-cells through a regenerative medicine approach has great potential as a therapeutic alternative to the traditional therapy of lifelong insulin injections.

*In vivo* transdifferentiation is a promising strategy whereby the initial cell source is abundant, and the immune rejection could be avoided. In 2008, the first documented *in vivo* direct reprogramming was performed of β-cells regeneration converted from pancreatic exocrine cells (acinar cells) via the combinatory expression of three transcription factors Ngn3, Pdx1 and MafA (Zhou et al., 2008; Li et al., 2014). Further investigation found this combination could also induce the gastrointestinal epithelial cells into endocrine β-like cells (Chen Y.-J. et al., 2014). Similarly, cells of the antral stomach also tend to complete this conversion (Ariyachet et al., 2016). Only the combination of Pdx1 and MafA delivered by adeno-associated virus (AAVs) through pancreatic duct could reprogram alpha cells into beta cells in mouse and human cells (Xiao et al., 2018; Furuyama et al., 2019). Moreover, by taking advantages of the close developmental relationship, ectopic expression of Tgif2 can convert hepatic cell fate to that of pancreatic progenitors (Cerda-Esteban et al., 2017). Unexpectedly, depletion of Foxo1 in Neurog3 (+) enteroendocrine progenitor cells generated mature β-like cells with insulin and C-peptide secreting capacity in response to glucose and sulfonylureas (Talchai et al., 2012). Additionally, the inactivation of Fbw7 could convert pancreatic ductal cells into α, δ, and β cells (Sancho et al., 2014) (Table 1).

**TABLE 1:** Cell reprogramming for Islet β-cells.

Factors	Methods	Starting Cell Type	Target Cell Type	Disease/Model	References
NGN3, PDX1, MAFA	overexpression	acinar cells	pancreatic β-cells	Type I diabetes	(Zhou et al., 2008; Li et al., 2014)
NGN3, PDX1, MAFA	overexpression	gastrointestinal epithelial cells	pancreatic β-cells	Type I diabetes	Chen et al. (2014b)
NGN3, PDX1, MAFA	overexpression	antral stomach cells	pancreatic β-cells	Type I diabetes	Ariyachet et al. (2016)
PDX1, MAFA	overexpression	pancreatic α-cells	pancreatic β-cells	Type I diabetes	(Xiao et al., 2018; Furuyama et al., 2019)
TGIF2	overexpression	hepatocytes	pancreatic progenitors	—	Cerda-Esteban et al. (2017)
FOXO1	ablation	Neurog3+ endocrine progenitor cells	pancreatic β-cells	Type I diabetes	Talchai et al. (2012)
FBW7	ablation	pancreatic ductal cells	pancreatic α-, δ-, and β- cells	—	Sancho et al. (2014)

However, one of the challenges in this field is that the newly induced β-cells cannot persist for long in type I diabetes due to autoimmunity (Pipeleers et al., 2002; Zaret and Grompe, 2008). Moreover, it has been reported that β-cells could be reactivated through the reversal of autoimmunity (Tang et al., 2020). Therefore, the combination of anti-autoimmune therapy would have a positive effect on the regeneration of β-cells (Wherrett et al., 2011; Bluestone et al., 2015; Wang et al., 2018; Tang et al., 2020). Alternatively, some porous biomaterials, like alginate derivatives, could be utilized as immune barrier to prevent these cells from the attack of autoimmunity (Vegas et al., 2016). Intriguingly, gene therapy through ectopic expression of Pdx1 and MafA by AAVs could obtain insulin-producing cells with the resistance of autoimmunity in the non-obese diabetic (NOD) mouse (Xiao et al., 2018).

Overall, these diverse approaches all show promises in generating functional cells, thus, providing solid foundations for future pre- and clinical development for curing type I and II diabetes with cell reprogramming.

## Cardiomyocytes

Heart failure is one of the most serious diseases that threaten human life in the modern era, affecting more than 14 million people worldwide (Laflamme and Murry, 2011; Qian et al., 2012; Arrigo et al., 2020; Roger, 2021; Savarese et al., 2022). About 1/4 cardiomyocytes (CMs) dysfunction a few hours after a myocardial infarction in the human left ventricle, but the left ventricle resident cardiomyocytes have little capability of proliferation after birth (Murry et al., 2006). Even with the aging process, the left and right ventricular myocardium have been estimated to lose 38 million and 14 million myocyte nuclei/year irreversibly, along with the enlargement of the remaining cardiomyocytes (Olivetti et al., 1991). Therefore, it is critical to obtain the abundant functional cardiomyocytes capable of repairing the injured or aging heart.

Several approaches have been reported. First, combinatorial expression of two cardiac transcription factors, Gata4 and Tbx5 and the subunit of BAF chromatin remodeling complexes, Smarcd3, has been reported to convert mouse mesoderm into cardiomyocytes efficiently (Takeuchi and Bruneau, 2009). Secondly, Gata4, Tbx5 and Mef2c can induce the direct reprogramming of postnatal cardiac or dermal fibroblasts into cardiomyocyte-like cells in a complementary approach (Ieda et al., 2010). Remarkably, this combination of the three transcription factors (Gata4, Tbx5 and Mef2c) with or without the fourth factor Hand2 can convert the postnatal murine cardiac fibroblasts into induced cardiomyocytes in the mouse model of myocardial infarction for the first time (Qian et al., 2012; Song et al., 2012). Mechanically, Mef2c and Tbx5 serve to open the compacted chromatin and the factors behave through context-specific cooperative mechanisms to guide cardiac reprogramming (Liu et al., 2020). Thirdly, miRNAs (miRNAs 1,133, 208, and 499) can convert fibroblasts to cardiomyocytes *in vitro* and *in vivo* (Jayawardena et al., 2012). Fourthly, a combination of microRNA mimic miR-208b-3p with the treatment of ascorbic acid and bone morphogenetic protein 4 (BMP4) can trigger the formation of a tissue-like structure containing three distinct cell types, cardiomyocytes, endothelial cells and smooth muscle cells from mouse tail-tip fibroblasts and this structure could be transplanted for the restoration of the infarcted hearts (Cho et al., 2021). Finally, nine chemicals (9C) can induce human fibroblasts to generate cardiomyocyte-like cells *in vivo* of mouse heart infarction model (Cao et al., 2016) (Table 2).

**TABLE 2:** Cell reprogramming for Cardiomyocytes.

Factors	Methods	Starting Cell Type	Target Cell Type	Disease/Model	References
TBX5, MEF2C, GATA4	overexpression	fibroblasts	cardiomyocytes	myocardial infarction	Qian et al. (2012)
TBX5, MEF2C, GATA4, HAND2	overexpression	fibroblasts	cardiomyocytes	myocardial infarction	Song et al. (2012)
OCT4, SOX2, KLF4, c-MYC	overexpression	adult cardiomyocytes	fetal cardiomyocytes	myocardial infarction	Chen et al. (2021)
Mir1133, 208, 499	overexpression	fibroblasts	cardiomyocytes	myocardial infarction	Jayawardena et al. (2012)
TBX5, MEF2C, GATA4, MESP1, MYOCD1	overexpression	human fibroblasts	cardiomyocyte-like cells	—	Wada et al. (2013)
	tissue formation	tail-tip fibroblasts	cardiomyocytes, endothelial cells, smooth muscle cells	myocardial infarction	Cho et al. (2021)
	chemical induction	fibroblasts	cardiomyocytes	myocardial infarction	Cao et al. (2016)
	chemical induction	fibroblasts	cardiovascular progenitor cells	myocardial infarction	Wang et al. (2022)

There were discrepancies associated with some of the above mentioned methods. For instance, while the three transcription factors Gata4, Mef2 and Tbx5 (GMT) can realize a robust transdifferentiation to cardiomyocytes in mouse, the same combination fails to perform the corresponding reprogramming in human (Yamakawa and Ieda, 2021). In the later research, Rie Wada and others achieved this purpose of transdifferentiation from human fibroblasts by addition of two cardiac-specific genes MESP1 and MYOCD (GMTMM) (Wada et al., 2013). Although other combinations, like six factors (Gata4, Tbx5, Hand2, Myocd, miR-1, and miR-133) (Nam et al., 2013) and seven factors (Gata4, Mef2c, Tbx5, Mesp1, Myocd, Esrrg, and Zfpm2) (Fu et al., 2013) have also been reported to complete this transition, almost all documented cases have lower efficiency of conversion comparing with that in mouse and lack functional beating property (Yamakawa and Ieda, 2021) (Table 2). These studies highlight the need to standardize protocols and criteria used in evaluating efficacy.

In addition to the transdifferentiation from cardiac fibroblasts into cardiomyocytes to alleviate the symptom of heat failure, enhancing the capability of myocardial regeneration was considered another promising strategy. Through the transient ectopic heart-specific expression of Oct4, Sox2, Klf4, and c-Myc (OSKM), the adult mouse cardiomyocytes could be reset into a kind of fetal state whereby nondividing cardiomyocytes regain their regenerative capacity (Chen et al., 2021). Notably, the long-term expression of OSKM tends to lead to tumor formation (Chen et al., 2021).

A newly isolated culture of expandable cardiovascular progenitor cells (CPCs) was reported with promising characteristics for regenerative medicine (Lalit et al., 2016; Zhang et al., 2016; Wang et al., 2022). These multipotent stem cells exist exclusively in the embryonic development and could subsequently generate heart tissue (Laugwitz et al., 2005). Recently, CPCs were reported to be obtained by treatment with six small molecules from mouse or human fibroblasts and cultured in chemical define and xeno-free conditions (Wang et al., 2022), which provides a new and abundant cell source for cardiac cell therapy and enhances the clinical application prospects of CPCs.

## Neurons

Neural degenerative diseases such as Parkinson’s disease and Alzheimer disease remain untreatable. Major efforts in regenerative medicine are directed towards these two degenerative diseases. Due to limited capability of self-renewal in the adult central nervous system and the highly organized complicated neural circuits make the neurodegeneration or nerve injuries one of the most intractable diseases to cure.

The non-neuronal macroglia include NG2 glia and astrocytes. NG2 cells are the progenitors of myelinating oligodendrocytes with capability of self-renewal and proliferation (Kang et al., 2010). Astrocytes are widespread in the central nervous system and contain neural differentiation potential under pathological conditions (Magnusson et al., 2014). These characteristics make glial cells the ideal cell sources to replenish the loss functional neurons in neural degenerative diseases or neural injuries (Wang H. et al., 2021). Nevertheless, it has proved to be difficult for traditional strategies to replace the compensatory glial cells or scars with functional neurons (Barker et al., 2018). Recently, the approaches of direct reprogramming of resident glial cells to functional neurons *in vivo* have shed light on the repair of the diseased nervous system.

Since 2013, several groups have proved the feasibility that the glial cells can be converted to functional new neurons through ectopic expression of neural master gene(s) by different adeno-associated virus (AAV) serotypes or retrovirus, independently (Guo et al., 2014; Matsuda et al., 2019; Chen et al., 2020; Zhou et al., 2020). Single factors including NEUROD1 (Guo et al., 2014; Chen et al., 2020), SOX2 (Niu et al., 2013; Tai et al., 2021) or ASCL1 (Liu Y. et al., 2015) is enough to accomplish this kind of transition. It is worth noting that SOX2 seems to induce the glial cells into a progenitor condition (Niu et al., 2013; Tai et al., 2021). Furthermore, this genetic method of transformation can be substituted by sequential exposure to a cocktail of small molecules *in vitro* (Zhang et al., 2015). Intriguingly, knocking down only one single RNA-binding protein, polypyrimidine tract-binding protein 1 (Ptbp1), which has been reported to induce the transdifferentiation of fibroblasts into neurons *in vitro* previously (Xue et al., 2013), can perform a robust conversion of astrocytes into functional dopaminergic neurons to alleviate motor defects in a Parkinson’s disease mouse model by CRISPR system CasRx (Zhou et al., 2020) or antisense oligonucleotides (ASOs) (Qian et al., 2020). Additionally, this approach can also alleviate the eyesight of retinal injury mouse model by reprogramming Müller glia into retinal ganglion cells (RGCs) (Zhou et al., 2020). If true, the small molecules targeting Ptbp1 may also be utilized in the design of therapeutics for neurodegeneration or nerve injuries (Table 3).

**TABLE 3:** Cell reprogramming for Neurons.

Factors	Methods	Starting Cell Type	Target Cell Type	Disease/Model	References
NEUROD1	overexpression	astrocytes	glutamatergic neurons	brain injury/Alzheimer’s disease	Guo et al. (2014)
		NG2 cells	GABAergic and glutamatergic neurons		
NEUROD1	overexpression	Microglia	Neurons	—	Matsuda et al. (2019)
NEUROD1	overexpression	astrocytes	Neurons	ischemic injury	Chen et al. (2020)
PTBP1	Knockdown by shRNA/ASOs	midbrain astrocytes	dopaminergic neurons	Parkinson’s disease	Qian et al. (2020)
PTBP1	Knockdown by CRISPR-CasRx	Müller glia	retinal ganglion cells (RGCs)	Retinal Injury	Zhou et al. (2020)
		striatal astrocytes	dopaminergic neurons	Parkinson’s disease	
SOX2	overexpression	astrocytes	neuroblasts	—	Niu et al. (2013)
SOX2	overexpression	NG2 glial cells	ASCL1+ progenitor cells	spinal cord injury (SCI)	Tai et al. (2021)
ASCL1	overexpression	astrocytes	Neurons	—	Liu et al. (2015b)
NEUROGENIN2	overexpression	non-neuronal cells	Neurons	—	Grande et al. (2013)
NURR1, NEUROGENE2	overexpression	astrocytes	Neurons	Cerebral Cortex Injury	Mattugini et al. (2019)
ASCL1, LMX1A, NURR1	overexpression	striatal NG2 glia	GABAergic and glutamatergic neurons	—	Torper et al. (2015)
NEURD1, DLX2	overexpression	striatal astrocytes	GABAergic neurons	Huntington’s disease	Wu et al. (2020)
ASCL1, NEUROD1, LMX1A, Mir218	overexpression	astrocytes	dopaminergic neurons	Parkinson’s disease	Rivetti di Val Cervo et al. (2017)
	chemical induction	astrocytes	Neurons	—	Zhang et al. (2015)

Nevertheless, the promising strategies of glia-to-neuron conversion have also been challenged by the work recently reported (Wang L.-L. et al., 2021; Rao et al., 2021). By rigorous lineage tracing methods and various AAVs, they questioned the capability of NeuroD1 of inducing the reprogramming from astrocytes to functional neurons. Instead, the so-called newborn neurons are the progenies of resident neurons. Furthermore, the astrocytes-to-neurons transition also fails to be detected upon the depletion of PTBP1 (Wang L.-L. et al., 2021). Meanwhile, the NeuroD1-induced microglia-to-neuron conversion is also questioned (Rao et al., 2021).

In summary, despite the reported induced reprogramming from the resident glial cells to functional neurons, this is a promising means to replenish the loss of neurons resulting from the neurodegenerative diseases or nerve injuries. The details underlying this process and the original cell species are still controversial, which is partly on account of the variant experimental conditions, including virus titer, the parameters and window of detection and the disease models to name a few. A broad and comprehensive knowledge will be necessary before we can perform the clinical trials.

## Tumor Cells

In theory, it should be possible to apply iPSC technology to tumor cells, to convert them to a benign phenotype, or one sensitive to chemotherapeutic drugs. Indeed, Utikal et al. identified that mouse malignant R545 melanoma cell line could be reprogrammed into iPSCs by the transfection of Yamanaka factors (Utikal et al., 2009). The expression of endogenous Oct4, Klf4, and Myc was activated in these iPSC clones derived from parental R545 cells. Besides, these iPSC lines also showed demethylation of the pluripotency-related gene Oct4 and Nanog promoters and the decrease of tumorigenicity *in vivo* (Utikal et al., 2009). In 2010, Mioshi et al. reported that gastrointestinal cancer cells, including pancreatic, liver, and colorectal cancer cells, acquired pluripotency so that these cells displayed the potential to form ectoderm, mesoderm, and endoderm morphological patterns after the introduction of Yamanaka factors. Induced cells were less malignant both *in vitro* and *in vivo* but showed higher sensitivity to 5-fluorodeoxyuridine than the parental cells (Miyoshi et al., 2010). To explore the effect of oncogene on cancer cell reprogramming, Carette et al. reprogrammed human KBM7 chronic myeloid leukemia cells, which harbored breakpoint-cluster region -Abelson leukemia gene (BCR-ABL) fusion, through the retrovirus-mediated expression of Yamanaka factors. They found that reprogramming activated the expression of pluripotency-related genes and restored the differentiation potency in cancer cell-derived iPSCs. In addition, reprogrammed cells lost dependency on BCR-ABL oncogene signaling and possessed resistance to the inhibitor treatment, leading to a potential approach for leukemia treatment (Carette et al., 2010). Similarly, in the study of Liu et al., human mixed-lineage leukemia-AF9 fusion gene overexpressed acute myeloid leukemia (AML) cells were successfully converted into iPSCs through Yamanaka factors transfection. Reprogramming re-wrote epigenetic patterns so that oncogenic gene MLL-AF9 was silenced (Liu et al., 2014). To investigate whether the acquired benign feature could maintain during terminal differentiation into initial and alternative cell lineages, human osteosarcoma, liposarcoma, and sarcomas were reprogrammed by transfection of OCT4, NANOG, SOX2, LIN28, KLF4, and MYC. Interestingly, each pattern of terminal differentiation of such induced cells could abrogate the tumorigenicity of parental cells (Zhang et al., 2013) (Table 4). *Cancer* cell reprogramming and subsequent differentiation might be a promising strategy for the treatment of cancer.

**TABLE 4:** Cell reprogramming from tumor cells.

Factors	Methods	Starting Cell Type	Target Cell Type	Disease/Model	References
Oct4, Sox2, Klf4, Myc	overexpression	R545	Pluripotent embryonic stem-like cells	Melanoma	Utikal et al. (2009)
OCT4, SOX2, KLK4, MYC	overexpression	PaCa-2, PLC, DLD-1, HCT116	Pluripotent embryonic stem-like cells	Pancreatic, liver, colon cancer	Miyoshi et al. (2010)
OCT4, SOX2, KLK4, MYC	overexpression	KBM7	Pluripotent embryonic stem-like cells	Leukemia	Carette et al. (2010)
OCT4, SOX2, Nanog, KLK4, MYC, LIN28A	overexpression	HOS, SAOS2, MG63 et al	Pluripotent embryonic stem-like cells	Osteosarcoma	Zhang et al. (2013)
OCT4, SOX2, KLK4, MYC	overexpression	Primary AML cells	Pluripotent embryonic stem-like cells	Leukemia	Liu et al. (2014)
OCT4, NANOG, SOX2, KLF4, MYC, MIR302A	overexpression	HCT116, HT29, DLD1 et al	Pluripotent embryonic stem-like cells	prostate, brain, breast cancer et al	Mathieu et al. (2011)

Despite of these reported studies, only a minority of cancer cells are amenable to successful reprogramming. One obvious reason is certain mutations and genomic instability might impede cancer cell reprogramming. For instance, it has proved that Notch1 mutation induced T-acute lymphoblastic leukemia could not be converted into the pluripotent state (Liu et al., 2014). Besides, the majority of cancer patients undergo chemo- or radio-therapy before surgical resection. Whether such treatments could prevent the generation of cancer cell-derived iPSCs remains unclear. Therefore, although such iPSC technology is feasible to reverse many types of cancer cells back into a pluripotent state with benign features, the efficiency, safety and universality remains the huge challenge for its clinical application.

## Principles of Cell Reprogramming

### Chromatin Accessibility Dynamics During Reprogramming

**FIGURE 1:**
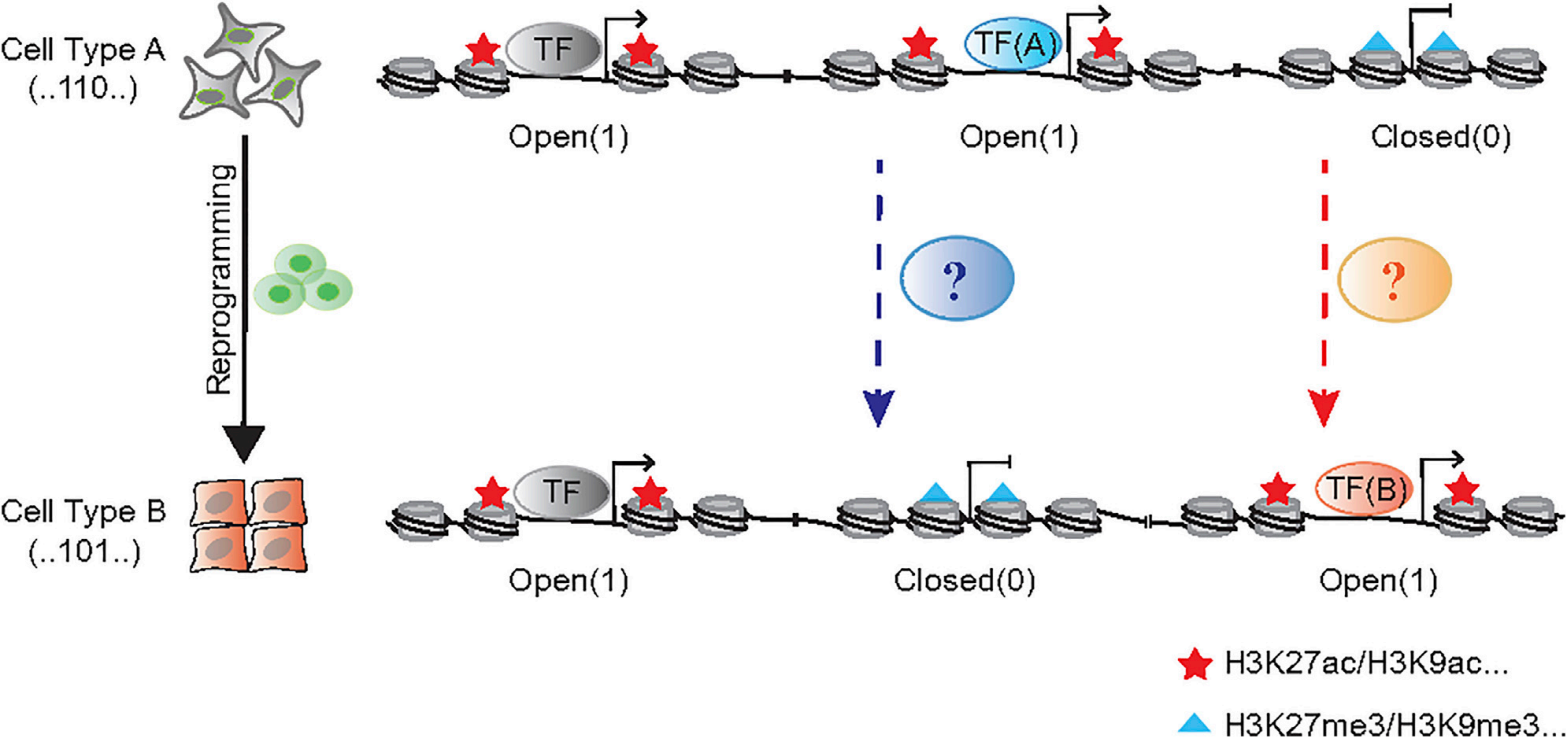
The binary chromatin accessibility code during cell reprogramming. The chromatin remodeler complexes, like BAF and NuRD function to open and close chromatin in the process of cell fate transition, respectively. The cellular morphological changes, such as EMT/MET, always accompany with that process.

The reprogramming approaches discussed above are quite diverse in terms of factors and cell types involved. One may wish to search for general principles governing these diverse processes. As it takes anywhere between 40 and 200 trillion cells to make a human, making up our brains, muscles, organs and every part of us (Bianconi et al., 2013). Yet, each of these cells share one common genome, thus, inspiring hope that unifying principles can be researched and identified. Now tools are becoming available at a faster pace, this may allow us to interrogate the genome in much greater detail. Indeed, recent cell atlas efforts have shown that each cell type possesses a unique chromatin accessibility pattern (Cusanovich et al., 2018; Cao et al., 2020; Zhang et al., 2021), which may serve as a fingerprint for the identification of cell types undergoing reprogramming or fate transition. To this end, our group have recently made progress in profiling chromatin accessibility dynamics (CAD) during reprogramming (Li D. et al., 2017; Cao et al., 2018). We have provided a general logic for understanding cell fate transition at the chromatin level as shown in Figure 1. If we mark the open chromatin sites by “1” and the opposite sites by “0” during the chromatin accessibility dynamics, every cell type could be labeled by a specific binary chromatin accessibility code.

It would be an intractable task to screen for the appropriate combination of factors from the highly variable gene list to perform the cell reprogramming into the targeted cell type. The analysis of chromatin accessibility dynamics could offer certain clues in that the core factors’ footprints would be uncovered from the dynamic sites. Directly comparing the chromatin accessibility dynamics between mESCs and mouse embryonic fibroblasts (MEFs) revealed that the most enriched binding motifs of transcription factors in the open sites were those of OCT, SOX2 and KLF (Li D. et al., 2017), i.e., the core Yamanaka factors in somatic cells reprogramming (Takahashi and Yamanaka, 2006). Additionally, the highest ranking enriched motifs within closed sites were the AP-1 family which would impede somatic cells reprogramming dramatically, and the AP-1 antagonist JDP2 could even substitute OCT4 to realize this transition (Liu J. et al., 2015). While the motif analysis of chromatin accessibility dynamics is an approach to rationally predict critical factors to perform unimplemented cell reprogramming, it must rely on pre-existing motif databases and the culture medium to capture the cell types of interest. Also, this method cannot be used to predict the essential regulators without DNA binding motif.

### The General Protein Machine During Reprogramming

The process of cellular reprogramming is invariably accompanied by reduction of the characteristics of initial cells and the acquisition of those in targeted cells, which is also revealed by the dynamic changes in genomic accessibility. Pioneer factors serve a pivotal role in facilitating chromatin accessibility when cell fate is converted, such as NEUROD1 in microglia-neuron transition (Matsuda et al., 2019), SOX2 in somatic cell reprogramming (Chen J. et al., 2014), FOXA in fibroblast-hepatocyte transdifferentiation (Huang et al., 2011; Sekiya and Suzuki, 2011). But generally, ATP-dependent chromatin remodeling complex BAF (BRG1/BRM-associated Factor) serves a broader role in exposing DNA that wraps around nucleosomes and opens chromatin, which participates almost all the biological process (Ho and Crabtree, 2010; Hodges et al., 2016; Cenik and Shilatifard, 2021). In addition to the enzyme core subunit BRG1/BRM, the recent works suggest the other accessory compositions are also involved in reprogramming regulation, like DPF2 (Zhang et al., 2019), SS18 (Kuang et al., 2021) and PBRM1 (Sinha et al., 2020). In contrast with the opening of chromatin during reprogramming, it is equally important to close the genomic loci representing the initial cell state. Histone deacetylase 1/2 (HDAC1/2) contained nucleosome remodeling and histone deacetylation (NuRD) complex could be one of the “brake” apparatuses (Li D. et al., 2017) (Figure 1).

### The Cell Morphological Changes During Reprogramming

In addition to the chromatin dynamics, cell morphological changes are also a phenomenon that cannot be ignored, which would provide certain guiding significance for the optimization in the process of cellular reprogramming. Mesenchymal-to-epithelial transitions (MET) or the opposite process EMT could provide a kind of that conceptual framework (Li et al., 2010; Zhao et al., 2015; Li Q. et al., 2017; Wang et al., 2022) to investigate the underlying mechanism and improve the strategy for reprogramming.

### Chemical Induction of Reprogramming

Cell reprogramming induced by the cocktail of small molecules alone holds huge promise in regenerative medicine in that the chemical compounds are convenient to be synthesized, preserved, and standardized, and the effects are often reversible (Yu et al., 2014; Qin et al., 2017). However, in the absence of directional lineage-specific factors, reprogramming process induced by small molecules tends to be context dependent with one or more intermediate stages whereby the cell fate is highly plastic and has the potential for multidirectional differentiation (Cao et al., 2017; Li X. et al., 2017; Cao et al., 2018; Guan et al., 2022). Unlike the transcription factors that activate downstream functional gene expression directly, small molecules regulate cell fate transition through interfering epigenetic machines, signal pathways, metabolic fluxes, etc., which results in a relatively less efficient and longer process (Yu et al., 2014; Qin et al., 2017).

## Conclusion and Perspectives

The successful obtainment of functional cells through cell reprogramming enables cell replacement therapy for regenerative medicine. In the last 4 decades, various cell types with no or little capability of regeneration, including cardiomyocytes, neurons, and Islet β-cells were acquired by direct or trans differentiation *in vivo* or *vitro* utilizing transcription factors, cytokines and/or small molecules.

As research on the cell atlas progresses, the increasing cell types are revealed, but only a minority of which we can control cell fate. To date, we still lack enough successful paradigms to draw a general principle/law to instruct the performance of cell reprogramming, especially for small molecules induced process. One of the well-studied barriers preventing the transition between different cell fate is the epigenetic landscape and the future promising work in this field is to figure out whether there are core epigenetic rules to stabilize cell fate and the universal tools to remove these barriers. Another thorny problem is that there are still very few cell types that can be cultured *in vitro*, which results in the failure to capture the target cell population. In addition to developing more suitable media, 3D cultures and *in vivo* reprogramming approaches may provide alternative solutions. Of course, the emerging technologies, including Cas9 screening (Black et al., 2020; Kuang et al., 2021), antibody libraries screening (Blanchard et al., 2017), Cas9-derived endogenous genes regulation tools (Grath and Dai, 2019; Russo et al., 2021), computational modelling (Letort et al., 2019; Kang and Li, 2020), etc., will also positively contribute to this field.

## References

[B1] Ariyachet C. Tovaglieri A. Xiang G. Lu J. Shah M. S. Richmond C. A. (2016). Reprogrammed Stomach Tissue as a Renewable Source of Functional β Cells for Blood Glucose Regulation. Cell. Stem Cell. 18, 410–421. 10.1016/j.stem.2016.01.003 PubMed Abstract | 10.1016/j.stem.2016.01.003 | Google Scholar 26908146 PMC4779391

[B2] Arrigo M. Jessup M. Mullens W. Reza N. Shah A. M. Sliwa K. (2020). Acute Heart Failure. Nat. Rev. Dis. Prim. 6, 16. 10.1038/s41572-020-0151-7 PubMed Abstract | 10.1038/s41572-020-0151-7 | Google Scholar 32139695 PMC7714436

[B3] Bar-Nur O. Gerli M. F. M. Di Stefano B. Almada A. E. Galvin A. Coffey A. (2018). Direct Reprogramming of Mouse Fibroblasts into Functional Skeletal Muscle Progenitors. Stem Cell. Rep. 10, 1505–1521. 10.1016/j.stemcr.2018.04.009 PubMed Abstract | 10.1016/j.stemcr.2018.04.009 | Google Scholar PMC5995754 29742392

[B4] Barker R. A. Götz M. Parmar M. (2018). New Approaches for Brain Repair-From Rescue to Reprogramming. Nature 557, 329–334. 10.1038/s41586-018-0087-1 PubMed Abstract | 10.1038/s41586-018-0087-1 | Google Scholar 29769670

[B5] Bianconi E. Piovesan A. Facchin F. Beraudi A. Casadei R. Frabetti F. (2013). An Estimation of the Number of Cells in the Human Body. Ann. Hum. Biol. 40, 463–471. 10.3109/03014460.2013.807878 PubMed Abstract | 10.3109/03014460.2013.807878 | Google Scholar 23829164

[B6] Black J. B. McCutcheon S. R. Dube S. Barrera A. Klann T. S. Rice G. A. (2020). Master Regulators and Cofactors of Human Neuronal Cell Fate Specification Identified by CRISPR Gene Activation Screens. Cell. Rep. 33, 108460. 10.1016/j.celrep.2020.108460 PubMed Abstract | 10.1016/j.celrep.2020.108460 | Google Scholar 33264623 PMC7730023

[B7] Blanchard J. W. Xie J. El-Mecharrafie N. Gross S. Lee S. Lerner R. A. (2017). Replacing Reprogramming Factors with Antibodies Selected from Combinatorial Antibody Libraries. Nat. Biotechnol. 35, 960–968. 10.1038/nbt.3963 PubMed Abstract | 10.1038/nbt.3963 | Google Scholar 28892074 PMC12989212

[B8] Bluestone J. A. Buckner J. H. Fitch M. Gitelman S. E. Gupta S. Hellerstein M. K. (2015). Type 1 Diabetes Immunotherapy Using Polyclonal Regulatory T Cells. Sci. Transl. Med. 7, 315ra189. 10.1126/scitranslmed.aad4134 PubMed Abstract | 10.1126/scitranslmed.aad4134 | Google Scholar PMC4729454 26606968

[B9] Cao J. O'Day D. R. Pliner H. A. Kingsley P. D. Deng M. Daza R. M. (2020). A Human Cell Atlas of Fetal Gene Expression. Science 370, eaba7721. 10.1126/science.aba7721 PubMed Abstract | 10.1126/science.aba7721 | Google Scholar 33184181 PMC7780123

[B10] Cao N. Huang Y. Zheng J. Spencer C. I. Zhang Y. Fu J.-D. (2016). Conversion of Human Fibroblasts into Functional Cardiomyocytes by Small Molecules. Science 352, 1216–1220. 10.1126/science.aaf1502 PubMed Abstract | 10.1126/science.aaf1502 | Google Scholar 27127239

[B11] Cao S. Yu S. Chen Y. Wang X. Zhou C. Liu Y. (2017). Chemical Reprogramming of Mouse Embryonic and Adult Fibroblast into Endoderm Lineage. J. Biol. Chem. 292, 19122–19132. 10.1074/jbc.m117.812537 PubMed Abstract | 10.1074/jbc.m117.812537 | Google Scholar 28935668 PMC5704493

[B12] Cao S. Yu S. Li D. Ye J. Yang X. Li C. (2018). Chromatin Accessibility Dynamics during Chemical Induction of Pluripotency. Cell. Stem Cell. 22, 529–542. e525. 10.1016/j.stem.2018.03.005 PubMed Abstract | 10.1016/j.stem.2018.03.005 | Google Scholar 29625068

[B13] Carette J. E. Pruszak J. Varadarajan M. Blomen V. A. Gokhale S. Camargo F. D. (2010). Generation of iPSCs from Cultured Human Malignant Cells. Blood 115, 4039–4042. 10.1182/blood-2009-07-231845 PubMed Abstract | 10.1182/blood-2009-07-231845 | Google Scholar 20233975 PMC2875096

[B14] Carrió E. Magli A. Muñoz M. Peinado M. A. Perlingeiro R. Suelves M. (2016). Muscle Cell Identity Requires Pax7-Mediated Lineage-specific DNA Demethylation. BMC Biol. 14, 30. 10.1186/s12915-016-0250-9 PubMed Abstract | 10.1186/s12915-016-0250-9 | Google Scholar 27075038 PMC4831197

[B15] Cenik B. K. Shilatifard A. (2021). COMPASS and SWI/SNF Complexes in Development and Disease. Nat. Rev. Genet. 22, 38–58. 10.1038/s41576-020-0278-0 PubMed Abstract | 10.1038/s41576-020-0278-0 | Google Scholar 32958894

[B16] Cerdá-Esteban N. Naumann H. Ruzittu S. Mah N. Pongrac I. M. Cozzitorto C. (2017). Stepwise Reprogramming of Liver Cells to a Pancreas Progenitor State by the Transcriptional Regulator Tgif2. Nat. Commun. 8, 14127. 10.1038/ncomms14127 PubMed Abstract | 10.1038/ncomms14127 | Google Scholar 28193997 PMC5316826

[B17] Chal J. Pourquié O. (2017). Making Muscle: Skeletal Myogenesis *In Vivo* and *In Vitro* . Development 144, 2104–2122. 10.1242/dev.151035 PubMed Abstract | 10.1242/dev.151035 | Google Scholar 28634270

[B18] Chen J. Zhang Z. Li L. Chen B.-C. Revyakin A. Hajj B. (2014a). Single-molecule Dynamics of Enhanceosome Assembly in Embryonic Stem Cells. Cell. 156, 1274–1285. 10.1016/j.cell.2014.01.062 PubMed Abstract | 10.1016/j.cell.2014.01.062 | Google Scholar 24630727 PMC4040518

[B19] Chen Y.-C. Ma N.-X. Pei Z.-F. Wu Z. Do-Monte F. H. Keefe S. (2020). A NeuroD1 AAV-Based Gene Therapy for Functional Brain Repair after Ischemic Injury through *In Vivo* Astrocyte-To-Neuron Conversion. Mol. Ther. 28, 217–234. 10.1016/j.ymthe.2019.09.003 PubMed Abstract | 10.1016/j.ymthe.2019.09.003 | Google Scholar 31551137 PMC6952185

[B20] Chen Y.-J. Finkbeiner S. R. Weinblatt D. Emmett M. J. Tameire F. Yousefi M. (2014b). De Novo Formation of Insulin-Producing "Neo-β Cell Islets" from Intestinal Crypts. Cell. Rep. 6, 1046–1058. 10.1016/j.celrep.2014.02.013 PubMed Abstract | 10.1016/j.celrep.2014.02.013 | Google Scholar 24613355 PMC4245054

[B21] Chen Y. Lüttmann F. F. Schoger E. Schöler H. R. Zelarayán L. C. Kim K.-P. (2021). Reversible Reprogramming of Cardiomyocytes to a Fetal State Drives Heart Regeneration in Mice. Science 373, 1537–1540. 10.1126/science.abg5159 PubMed Abstract | 10.1126/science.abg5159 | Google Scholar 34554778

[B22] Cho J. Kim S. Lee H. Rah W. Cho H. C. Kim N. K. (2021). Regeneration of Infarcted Mouse Hearts by Cardiovascular Tissue Formed via the Direct Reprogramming of Mouse Fibroblasts. Nat. Biomed. Eng. 5, 880–896. 10.1038/s41551-021-00783-0 PubMed Abstract | 10.1038/s41551-021-00783-0 | Google Scholar 34426676 PMC8809198

[B23] Constantinides P. G. Jones P. A. Gevers W. (1977). Functional Striated Muscle Cells from Non-myoblast Precursors Following 5-azacytidine Treatment. Nature 267, 364–366. 10.1038/267364a0 PubMed Abstract | 10.1038/267364a0 | Google Scholar 68440

[B24] Cusanovich D. A. Hill A. J. Aghamirzaie D. Daza R. M. Pliner H. A. Berletch J. B. (2018). A Single-Cell Atlas of *In Vivo* Mammalian Chromatin Accessibility. Cell. 174, 1309–1324. 10.1016/j.cell.2018.06.052 PubMed Abstract | 10.1016/j.cell.2018.06.052 | Google Scholar 30078704 PMC6158300

[B25] Darabi R. Arpke R. W. Irion S. Dimos J. T. Grskovic M. Kyba M. (2012). Human ES- and iPS-Derived Myogenic Progenitors Restore DYSTROPHIN and Improve Contractility upon Transplantation in Dystrophic Mice. Cell. Stem Cell. 10, 610–619. 10.1016/j.stem.2012.02.015 PubMed Abstract | 10.1016/j.stem.2012.02.015 | Google Scholar 22560081 PMC3348507

[B26] Darabi R. Gehlbach K. Bachoo R. M. Kamath S. Osawa M. Kamm K. E. (2008). Functional Skeletal Muscle Regeneration from Differentiating Embryonic Stem Cells. Nat. Med. 14, 134–143. 10.1038/nm1705 PubMed Abstract | 10.1038/nm1705 | Google Scholar 18204461

[B27] Davis R. L. Weintraub H. Lassar A. B. (1987). Expression of a Single Transfected cDNA Converts Fibroblasts to Myoblasts. Cell. 51, 987–1000. 10.1016/0092-8674(87)90585-x PubMed Abstract | 10.1016/0092-8674(87)90585-x | Google Scholar 3690668

[B28] Fu J.-D. Srivastava D. (2015). Direct Reprogramming of Fibroblasts into Cardiomyocytes for Cardiac Regenerative Medicine. Circ. J. 79, 245–254. 10.1253/circj.cj-14-1372 PubMed Abstract | 10.1253/circj.cj-14-1372 | Google Scholar 25744738

[B29] Fu J.-D. Stone N. R. Liu L. Spencer C. I. Qian L. Hayashi Y. (2013). Direct Reprogramming of Human Fibroblasts toward a Cardiomyocyte-like State. Stem Cell. Rep. 1, 235–247. 10.1016/j.stemcr.2013.07.005 10.1016/j.stemcr.2013.07.005 | Google Scholar PMC3849259 24319660

[B30] Furuyama K. Chera S. van Gurp L. Oropeza D. Ghila L. Damond N. (2019). Diabetes Relief in Mice by Glucose-Sensing Insulin-Secreting Human α-cells. Nature 567, 43–48. 10.1038/s41586-019-0942-8 PubMed Abstract | 10.1038/s41586-019-0942-8 | Google Scholar 30760930 PMC6624841

[B31] Gascón S. Masserdotti G. Russo G. L. Götz M. (2017). Direct Neuronal Reprogramming: Achievements, Hurdles, and New Roads to Success. Cell. Stem Cell. 21, 18–34. 10.1016/j.stem.2017.06.011 PubMed Abstract | 10.1016/j.stem.2017.06.011 | Google Scholar 28686866

[B32] Ge J. Liu K. Niu W. Chen M. Wang M. Xue Y. (2018). Gold and Gold-Silver Alloy Nanoparticles Enhance the Myogenic Differentiation of Myoblasts through P38 MAPK Signaling Pathway and Promote *In Vivo* Skeletal Muscle Regeneration. Biomaterials 175, 19–29. 10.1016/j.biomaterials.2018.05.027 PubMed Abstract | 10.1016/j.biomaterials.2018.05.027 | Google Scholar 29793089

[B33] Goudenege S. Lebel C. Huot N. B. Dufour C. Fujii I. Gekas J. (2012). Myoblasts Derived from Normal hESCs and Dystrophic hiPSCs Efficiently Fuse with Existing Muscle Fibers Following Transplantation. Mol. Ther. 20, 2153–2167. 10.1038/mt.2012.188 PubMed Abstract | 10.1038/mt.2012.188 | Google Scholar 22990676 PMC3498803

[B34] Grande A. Sumiyoshi K. López-Juárez A. Howard J. Sakthivel B. Aronow B. (2013). Environmental Impact on Direct Neuronal Reprogramming *In Vivo* in the Adult Brain. Nat. Commun. 4, 2373. 10.1038/ncomms3373 PubMed Abstract | 10.1038/ncomms3373 | Google Scholar 23974433 PMC3786770

[B35] Grath A. Dai G. (2019). Direct Cell Reprogramming for Tissue Engineering and Regenerative Medicine. J. Biol. Eng. 13, 14. 10.1186/s13036-019-0144-9 PubMed Abstract | 10.1186/s13036-019-0144-9 | Google Scholar 30805026 PMC6373087

[B36] Guan J. Wang G. Wang J. Zhang Z. Fu Y. Cheng L. (2022). Chemical Reprogramming of Human Somatic Cells to Pluripotent Stem Cells. Nature 605, 325–331. 10.1038/s41586-022-04593-5 PubMed Abstract | 10.1038/s41586-022-04593-5 | Google Scholar 35418683

[B37] Guo Z. Zhang L. Wu Z. Chen Y. Wang F. Chen G. (2014). *In Vivo* direct Reprogramming of Reactive Glial Cells into Functional Neurons after Brain Injury and in an Alzheimer's Disease Model. Cell. Stem Cell. 14, 188–202. 10.1016/j.stem.2013.12.001 PubMed Abstract | 10.1016/j.stem.2013.12.001 | Google Scholar 24360883 PMC3967760

[B38] Gurdon J. B. (1962). The Developmental Capacity of Nuclei Taken from Intestinal Epithelium Cells of Feeding Tadpoles. J. Embryol. Exp. Morphol. 10, 622–640. 10.1242/dev.10.4.622 PubMed Abstract | 10.1242/dev.10.4.622 | Google Scholar 13951335

[B39] Ho L. Crabtree G. R. (2010). Chromatin Remodelling during Development. Nature 463, 474–484. 10.1038/nature08911 PubMed Abstract | 10.1038/nature08911 | Google Scholar 20110991 PMC3060774

[B40] Hodges C. Kirkland J. G. Crabtree G. R. (2016). The Many Roles of BAF (mSWI/SNF) and PBAF Complexes in Cancer. Cold Spring Harb. Perspect. Med. 6, a026930. 10.1101/cshperspect.a026930 PubMed Abstract | 10.1101/cshperspect.a026930 | Google Scholar 27413115 PMC4968166

[B41] Huang P. He Z. Ji S. Sun H. Xiang D. Liu C. (2011). Induction of Functional Hepatocyte-like Cells from Mouse Fibroblasts by Defined Factors. Nature 475, 386–389. 10.1038/nature10116 PubMed Abstract | 10.1038/nature10116 | Google Scholar 21562492

[B42] Ieda M. Fu J.-D. Delgado-Olguin P. Vedantham V. Hayashi Y. Bruneau B. G. (2010). Direct Reprogramming of Fibroblasts into Functional Cardiomyocytes by Defined Factors. Cell. 142, 375–386. 10.1016/j.cell.2010.07.002 PubMed Abstract | 10.1016/j.cell.2010.07.002 | Google Scholar 20691899 PMC2919844

[B43] Incitti T. Magli A. Darabi R. Yuan C. Lin K. Arpke R. W. (2019). Pluripotent Stem Cell-Derived Myogenic Progenitors Remodel Their Molecular Signature upon *In Vivo* Engraftment. Proc. Natl. Acad. Sci. U.S.A. 116, 4346–4351. 10.1073/pnas.1808303116 PubMed Abstract | 10.1073/pnas.1808303116 | Google Scholar 30760602 PMC6410870

[B44] Jayawardena T. M. Egemnazarov B. Finch E. A. Zhang L. Payne J. A. Pandya K. (2012). MicroRNA-mediated *In Vitro* and *In Vivo* Direct Reprogramming of Cardiac Fibroblasts to Cardiomyocytes. Circ. Res. 110, 1465–1473. 10.1161/circresaha.112.269035 PubMed Abstract | 10.1161/circresaha.112.269035 | Google Scholar 22539765 PMC3380624

[B45] Kang S. H. Fukaya M. Yang J. K. Rothstein J. D. Bergles D. E. (2010). NG2+ CNS Glial Progenitors Remain Committed to the Oligodendrocyte Lineage in Postnatal Life and Following Neurodegeneration. Neuron 68, 668–681. 10.1016/j.neuron.2010.09.009 PubMed Abstract | 10.1016/j.neuron.2010.09.009 | Google Scholar 21092857 PMC2989827

[B46] Kang X. Li C. (2020). Landscape Inferred from Gene Expression Data Governs Pluripotency in Embryonic Stem Cells. Comput. Struct. Biotechnol. J. 18, 366–374. 10.1016/j.csbj.2020.02.004 PubMed Abstract | 10.1016/j.csbj.2020.02.004 | Google Scholar 32128066 PMC7044515

[B47] Kuang J. Zhai Z. Li P. Shi R. Guo W. Yao Y. (2021). SS18 Regulates Pluripotent-Somatic Transition through Phase Separation. Nat. Commun. 12, 4090. 10.1038/s41467-021-24373-5 PubMed Abstract | 10.1038/s41467-021-24373-5 | Google Scholar 34215745 PMC8253816

[B48] Laflamme M. A. Murry C. E. (2011). Heart Regeneration. Nature 473, 326–335. 10.1038/nature10147 PubMed Abstract | 10.1038/nature10147 | Google Scholar 21593865 PMC4091722

[B49] Lalit P. A. Salick M. R. Nelson D. O. Squirrell J. M. Shafer C. M. Patel N. G. (2016). Lineage Reprogramming of Fibroblasts into Proliferative Induced Cardiac Progenitor Cells by Defined Factors. Cell. Stem Cell. 18, 354–367. 10.1016/j.stem.2015.12.001 PubMed Abstract | 10.1016/j.stem.2015.12.001 | Google Scholar 26877223 PMC4779406

[B50] Laugwitz K.-L. Moretti A. Lam J. Gruber P. Chen Y. Woodard S. (2005). Postnatal Isl1+ Cardioblasts Enter Fully Differentiated Cardiomyocyte Lineages. Nature 433, 647–653. 10.1038/nature03215 PubMed Abstract | 10.1038/nature03215 | Google Scholar 15703750 PMC5578466

[B51] Letort G. Montagud A. Stoll G. Heiland R. Barillot E. Macklin P. (2019). PhysiBoSS: a Multi-Scale Agent-Based Modelling Framework Integrating Physical Dimension and Cell Signalling. Bioinformatics 35, 1188–1196. 10.1093/bioinformatics/bty766 PubMed Abstract | 10.1093/bioinformatics/bty766 | Google Scholar 30169736 PMC6449758

[B52] Li D. Liu J. Yang X. Zhou C. Guo J. Wu C. (2017a). Chromatin Accessibility Dynamics during iPSC Reprogramming. Cell. Stem Cell. 21, 819–833. e816. 10.1016/j.stem.2017.10.012 PubMed Abstract | 10.1016/j.stem.2017.10.012 | Google Scholar 29220666

[B53] Li Q. Hutchins A. P. Chen Y. Li S. Shan Y. Liao B. (2017b). A Sequential EMT-MET Mechanism Drives the Differentiation of Human Embryonic Stem Cells towards Hepatocytes. Nat. Commun. 8, 15166. 10.1038/ncomms15166 PubMed Abstract | 10.1038/ncomms15166 | Google Scholar 28466868 PMC5418622

[B54] Li R. Liang J. Ni S. Zhou T. Qing X. Li H. (2010). A Mesenchymal-To-Epithelial Transition Initiates and Is Required for the Nuclear Reprogramming of Mouse Fibroblasts. Cell. Stem Cell. 7, 51–63. 10.1016/j.stem.2010.04.014 PubMed Abstract | 10.1016/j.stem.2010.04.014 | Google Scholar 20621050

[B55] Li W. Cavelti-Weder C. Zhang Y. Clement K. Donovan S. Gonzalez G. (2014). Long-term Persistence and Development of Induced Pancreatic Beta Cells Generated by Lineage Conversion of Acinar Cells. Nat. Biotechnol. 32, 1223–1230. 10.1038/nbt.3082 PubMed Abstract | 10.1038/nbt.3082 | Google Scholar 25402613

[B56] Li X. Liu D. Ma Y. Du X. Jing J. Wang L. (2017c). Direct Reprogramming of Fibroblasts via a Chemically Induced XEN-like State. Cell. Stem Cell. 21, 264–273. 10.1016/j.stem.2017.05.019 PubMed Abstract | 10.1016/j.stem.2017.05.019 | Google Scholar 28648365

[B57] Li Y. Li N. Ge J. Xue Y. Niu W. Chen M. (2019). Biodegradable Thermal Imaging-Tracked Ultralong Nanowire-Reinforced Conductive Nanocomposites Elastomers with Intrinsical Efficient Antibacterial and Anticancer Activity for Enhanced Biomedical Application Potential. Biomaterials 201, 68–76. 10.1016/j.biomaterials.2019.02.013 PubMed Abstract | 10.1016/j.biomaterials.2019.02.013 | Google Scholar 30798021

[B58] Liu J. Han Q. Peng T. Peng M. Wei B. Li D. (2015a). The Oncogene C-Jun Impedes Somatic Cell Reprogramming. Nat. Cell. Biol. 17, 856–867. 10.1038/ncb3193 PubMed Abstract | 10.1038/ncb3193 | Google Scholar 26098572

[B59] Liu M. Wang Y. Nie Z. Gai J. Bhat J. A. Kong J. (2020). Double Mutation of Two Homologous Genes YL1 and YL2 Results in a Leaf Yellowing Phenotype in Soybean [Glycine Max (L.) Merr]. Plant Mol. Biol. 103, 527–543. 10.1007/s11103-020-01008-9 PubMed Abstract | 10.1007/s11103-020-01008-9 | Google Scholar 32323129

[B60] Liu Y. Cheng H. Gao S. Lu X. He F. Hu L. (2014). Reprogramming of MLL-AF9 Leukemia Cells into Pluripotent Stem Cells. Leukemia 28, 1071–1080. 10.1038/leu.2013.304 PubMed Abstract | 10.1038/leu.2013.304 | Google Scholar 24150221 PMC4017259

[B61] Liu Y. Miao Q. Yuan J. Han S. Zhang P. Li S. (2015b). Ascl1 Converts Dorsal Midbrain Astrocytes into Functional Neurons *In Vivo* . J. Neurosci. 35, 9336–9355. 10.1523/jneurosci.3975-14.2015 PubMed Abstract | 10.1523/jneurosci.3975-14.2015 | Google Scholar 26109658 PMC6605193

[B62] Magnusson J. P. Göritz C. Tatarishvili J. Dias D. O. Smith E. M. K. Lindvall O. (2014). A Latent Neurogenic Program in Astrocytes Regulated by Notch Signaling in the Mouse. Science 346, 237–241. 10.1126/science.346.6206.237 PubMed Abstract | 10.1126/science.346.6206.237 | Google Scholar 25301628

[B63] Mathieu J. Zhang Z. Zhou W. Wang A. J. Heddleston J. M. Pinna C. M. A. (2011). HIF Induces Human Embryonic Stem Cell Markers in Cancer Cells. Cancer Res. 71, 4640–4652. 10.1158/0008-5472.can-10-3320 PubMed Abstract | 10.1158/0008-5472.can-10-3320 | Google Scholar 21712410 PMC3129496

[B64] Matsuda T. Irie T. Katsurabayashi S. Hayashi Y. Nagai T. Hamazaki N. (2019). Pioneer Factor NeuroD1 Rearranges Transcriptional and Epigenetic Profiles to Execute Microglia-Neuron Conversion. Neuron 101, 472–485. 10.1016/j.neuron.2018.12.010 PubMed Abstract | 10.1016/j.neuron.2018.12.010 | Google Scholar 30638745

[B65] Mattugini N. Bocchi R. Scheuss V. Russo G. L. Torper O. Lao C. L. (2019). Inducing Different Neuronal Subtypes from Astrocytes in the Injured Mouse Cerebral Cortex. Neuron 103, 1086–1095. 10.1016/j.neuron.2019.08.009 PubMed Abstract | 10.1016/j.neuron.2019.08.009 | Google Scholar 31488328 PMC6859713

[B66] Miyoshi N. Ishii H. Nagai K.-i. Hoshino H. Mimori K. Tanaka F. (2010). Defined Factors Induce Reprogramming of Gastrointestinal Cancer Cells. Proc. Natl. Acad. Sci. U.S.A. 107, 40–45. 10.1073/pnas.0912407107 PubMed Abstract | 10.1073/pnas.0912407107 | Google Scholar 20018687 PMC2806714

[B67] Murry C. E. Reinecke H. Pabon L. M. (2006). Regeneration Gaps. J. Am. Coll. Cardiol. 47, 1777–1785. 10.1016/j.jacc.2006.02.002 PubMed Abstract | 10.1016/j.jacc.2006.02.002 | Google Scholar 16682301

[B68] Nam Y.-J. Song K. Luo X. Daniel E. Lambeth K. West K. (2013). Reprogramming of Human Fibroblasts toward a Cardiac Fate. Proc. Natl. Acad. Sci. U.S.A. 110, 5588–5593. 10.1073/pnas.1301019110 PubMed Abstract | 10.1073/pnas.1301019110 | Google Scholar 23487791 PMC3619357

[B69] Niu W. Zang T. Zou Y. Fang S. Smith D. K. Bachoo R. (2013). *In Vivo* reprogramming of Astrocytes to Neuroblasts in the Adult Brain. Nat. Cell. Biol. 15, 1164–1175. 10.1038/ncb2843 PubMed Abstract | 10.1038/ncb2843 | Google Scholar 24056302 PMC3867822

[B70] Olivetti G. Melissari M. Capasso J. M. Anversa P. (1991). Cardiomyopathy of the Aging Human Heart. Myocyte Loss and Reactive Cellular Hypertrophy. Circ. Res. 68, 1560–1568. 10.1161/01.res.68.6.1560 PubMed Abstract | 10.1161/01.res.68.6.1560 | Google Scholar 2036710

[B71] Pipeleers D. Keymeulen B. Chatenoud L. Hendrieckx C. Ling Z. Mathieu C. (2002). A View on Beta Cell Transplantation in Diabetes. Ann. N. Y. Acad. Sci. 958, 69–76. 10.1111/j.1749-6632.2002.tb02948.x PubMed Abstract | 10.1111/j.1749-6632.2002.tb02948.x | Google Scholar 12021085

[B72] Qian H. Kang X. Hu J. Zhang D. Liang Z. Meng F. (2020). Reversing a Model of Parkinson's Disease with *In Situ* Converted Nigral Neurons. Nature 582, 550–556. 10.1038/s41586-020-2388-4 PubMed Abstract | 10.1038/s41586-020-2388-4 | Google Scholar 32581380 PMC7521455

[B73] Qian L. Huang Y. Spencer C. I. Foley A. Vedantham V. Liu L. (2012). *In Vivo* reprogramming of Murine Cardiac Fibroblasts into Induced Cardiomyocytes. Nature 485, 593–598. 10.1038/nature11044 PubMed Abstract | 10.1038/nature11044 | Google Scholar 22522929 PMC3369107

[B74] Qin H. Zhao A. Fu X. (2017). Small Molecules for Reprogramming and Transdifferentiation. Cell. Mol. Life Sci. 74, 3553–3575. 10.1007/s00018-017-2586-x PubMed Abstract | 10.1007/s00018-017-2586-x | Google Scholar 28698932 PMC11107793

[B75] Quattrocelli M. Swinnen M. Giacomazzi G. Camps J. Barthélemy I. Ceccarelli G. (2015). Mesodermal iPSC-Derived Progenitor Cells Functionally Regenerate Cardiac and Skeletal Muscle. J. Clin. Investig. 125, 4463–4482. 10.1172/jci82735 PubMed Abstract | 10.1172/jci82735 | Google Scholar 26571398 PMC4665797

[B76] Rao Y. Du S. Yang B. Wang Y. Li Y. Li R. (2021). NeuroD1 Induces Microglial Apoptosis and Cannot Induce Microglia-To-Neuron Cross-Lineage Reprogramming. Neuron 109, 4094–4108. e4095. 10.1016/j.neuron.2021.11.008 PubMed Abstract | 10.1016/j.neuron.2021.11.008 | Google Scholar 34875233

[B77] Rivetti di Val Cervo P. Romanov R. A. Spigolon G. Masini D. Martín-Montañez E. Toledo E. M. (2017). Induction of Functional Dopamine Neurons from Human Astrocytes *In Vitro* and Mouse Astrocytes in a Parkinson's Disease Model. Nat. Biotechnol. 35, 444–452. 10.1038/nbt.3835 PubMed Abstract | 10.1038/nbt.3835 | Google Scholar 28398344

[B78] Roger V. L. (2021). Epidemiology of Heart Failure. Circ. Res. 128, 1421–1434. 10.1161/circresaha.121.318172 PubMed Abstract | 10.1161/circresaha.121.318172 | Google Scholar 33983838

[B79] Russo G. L. Sonsalla G. Natarajan P. Breunig C. T. Bulli G. Merl-Pham J. (2021). CRISPR-mediated Induction of Neuron-Enriched Mitochondrial Proteins Boosts Direct Glia-To-Neuron Conversion. Cell. Stem Cell. 28, 524–534. 10.1016/j.stem.2020.10.015 PubMed Abstract | 10.1016/j.stem.2020.10.015 | Google Scholar 33202244 PMC7939544

[B80] Saho S. Satoh H. Kondo E. Inoue Y. Yamauchi A. Murata H. (2016). Active Secretion of Dimerized S100A11 Induced by the Peroxisome in Mesothelioma Cells. Cancer Microenviron. 9, 93–105. 10.1007/s12307-016-0185-2 PubMed Abstract | 10.1007/s12307-016-0185-2 | Google Scholar 27334300 PMC5264658

[B81] Sancho R. Gruber R. Gu G. Behrens A. (2014). Loss of Fbw7 Reprograms Adult Pancreatic Ductal Cells into α, δ, and β Cells. Cell. Stem Cell. 15, 139–153. 10.1016/j.stem.2014.06.019 PubMed Abstract | 10.1016/j.stem.2014.06.019 | Google Scholar 25105579 PMC4136739

[B82] Savarese G. Stolfo D. Sinagra G. Lund L. H. (2022). Heart Failure with Mid-range or Mildly Reduced Ejection Fraction. Nat. Rev. Cardiol. 19, 100–116. 10.1038/s41569-021-00605-5 PubMed Abstract | 10.1038/s41569-021-00605-5 | Google Scholar 34489589 PMC8420965

[B83] Sekiya S. Suzuki A. (2011). Direct Conversion of Mouse Fibroblasts to Hepatocyte-like Cells by Defined Factors. Nature 475, 390–393. 10.1038/nature10263 PubMed Abstract | 10.1038/nature10263 | Google Scholar 21716291

[B84] Sinha S. Biswas M. Chatterjee S. S. Kumar S. Sengupta A. (2020). Pbrm1 Steers Mesenchymal Stromal Cell Osteolineage Differentiation by Integrating PBAF-dependent Chromatin Remodeling and BMP/TGF-β Signaling. Cell. Rep. 31, 107570. 10.1016/j.celrep.2020.107570 PubMed Abstract | 10.1016/j.celrep.2020.107570 | Google Scholar 32348751

[B85] Song K. Nam Y.-J. Luo X. Qi X. Tan W. Huang G. N. (2012). Heart Repair by Reprogramming Non-myocytes with Cardiac Transcription Factors. Nature 485, 599–604. 10.1038/nature11139 PubMed Abstract | 10.1038/nature11139 | Google Scholar 22660318 PMC3367390

[B86] Tai W. Wu W. Wang L.-L. Ni H. Chen C. Yang J. (2021). *In Vivo* reprogramming of NG2 Glia Enables Adult Neurogenesis and Functional Recovery Following Spinal Cord Injury. Cell. Stem Cell. 28, 923–937. 10.1016/j.stem.2021.02.009 PubMed Abstract | 10.1016/j.stem.2021.02.009 | Google Scholar 33675690 PMC8106641

[B87] Takahashi K. Tanabe K. Ohnuki M. Narita M. Ichisaka T. Tomoda K. (2007). Induction of Pluripotent Stem Cells from Adult Human Fibroblasts by Defined Factors. Cell. 131, 861–872. 10.1016/j.cell.2007.11.019 PubMed Abstract | 10.1016/j.cell.2007.11.019 | Google Scholar 18035408

[B88] Takahashi K. Yamanaka S. (2006). Induction of Pluripotent Stem Cells from Mouse Embryonic and Adult Fibroblast Cultures by Defined Factors. Cell. 126, 663–676. 10.1016/j.cell.2006.07.024 PubMed Abstract | 10.1016/j.cell.2006.07.024 | Google Scholar 16904174

[B89] Takeuchi J. K. Bruneau B. G. (2009). Directed Transdifferentiation of Mouse Mesoderm to Heart Tissue by Defined Factors. Nature 459, 708–711. 10.1038/nature08039 PubMed Abstract | 10.1038/nature08039 | Google Scholar 19396158 PMC2728356

[B90] Talchai C. Xuan S. Kitamura T. DePinho R. A. Accili D. (2012). Generation of Functional Insulin-Producing Cells in the Gut by Foxo1 Ablation. Nat. Genet. 44, 406–412. S401. 10.1038/ng.2215 PubMed Abstract | 10.1038/ng.2215 | Google Scholar 22406641 PMC3315609

[B91] Tang S. Zhang M. Zeng S. Huang Y. Qin M. Nasri U. (2020). Reversal of Autoimmunity by Mixed Chimerism Enables Reactivation of β Cells and Transdifferentiation of α Cells in Diabetic NOD Mice. Proc. Natl. Acad. Sci. U.S.A. 117, 31219–31230. 10.1073/pnas.2012389117 PubMed Abstract | 10.1073/pnas.2012389117 | Google Scholar 33229527 PMC7733788

[B92] Torper O. Ottosson D. R. Pereira M. Lau S. Cardoso T. Grealish S. (2015). *In Vivo* Reprogramming of Striatal NG2 Glia into Functional Neurons that Integrate into Local Host Circuitry. Cell. Rep. 12, 474–481. 10.1016/j.celrep.2015.06.040 PubMed Abstract | 10.1016/j.celrep.2015.06.040 | Google Scholar 26166567 PMC4521079

[B93] Utikal J. Maherali N. Kulalert W. Hochedlinger K. (2009). Sox2 Is Dispensable for the Reprogramming of Melanocytes and Melanoma Cells into Induced Pluripotent Stem Cells. J. Cell. Sci. 122, 3502–3510. 10.1242/jcs.054783 PubMed Abstract | 10.1242/jcs.054783 | Google Scholar 19723802 PMC2746132

[B94] Vegas A. J. Veiseh O. Gürtler M. Millman J. R. Pagliuca F. W. Bader A. R. (2016). Long-term Glycemic Control Using Polymer-Encapsulated Human Stem Cell-Derived Beta Cells in Immune-Competent Mice. Nat. Med. 22, 306–311. 10.1038/nm.4030 PubMed Abstract | 10.1038/nm.4030 | Google Scholar 26808346 PMC4825868

[B95] Vierbuchen T. Ostermeier A. Pang Z. P. Kokubu Y. Südhof T. C. Wernig M. (2010). Direct Conversion of Fibroblasts to Functional Neurons by Defined Factors. Nature 463, 1035–1041. 10.1038/nature08797 PubMed Abstract | 10.1038/nature08797 | Google Scholar 20107439 PMC2829121

[B96] Wada R. Muraoka N. Inagawa K. Yamakawa H. Miyamoto K. Sadahiro T. (2013). Induction of Human Cardiomyocyte-like Cells from Fibroblasts by Defined Factors. Proc. Natl. Acad. Sci. U.S.A. 110, 12667–12672. 10.1073/pnas.1304053110 PubMed Abstract | 10.1073/pnas.1304053110 | Google Scholar 23861494 PMC3732928

[B97] Wang H. Yang Y. Liu J. Qian L. (2021a). Direct Cell Reprogramming: Approaches, Mechanisms and Progress. Nat. Rev. Mol. Cell Biol. 22, 410–424. 10.1038/s41580-021-00335-z PubMed Abstract | 10.1038/s41580-021-00335-z | Google Scholar 33619373 PMC8161510

[B98] Wang J. Gu S. Liu F. Chen Z. Xu H. Liu Z. (2022). Reprogramming of Fibroblasts into Expandable Cardiovascular Progenitor Cells via Small Molecules in Xeno-free Conditions. Nat. Biomed. Eng. 6, 403–420. 10.1038/s41551-022-00865-7 PubMed Abstract | 10.1038/s41551-022-00865-7 | Google Scholar 35361933

[B99] Wang L.-L. Serrano C. Zhong X. Ma S. Zou Y. Zhang C.-L. (2021b). Revisiting Astrocyte to Neuron Conversion with Lineage Tracing *In Vivo* . Cell 184, 5465–5481. 10.1016/j.cell.2021.09.005 PubMed Abstract | 10.1016/j.cell.2021.09.005 | Google Scholar 34582787 PMC8526404

[B100] Wang Y. Dorrell C. Naugler W. E. Heskett M. Spellman P. Li B. (2018). Long-Term Correction of Diabetes in Mice by *In Vivo* Reprogramming of Pancreatic Ducts. Mol. Ther. 26, 1327–1342. 10.1016/j.ymthe.2018.02.014 PubMed Abstract | 10.1016/j.ymthe.2018.02.014 | Google Scholar 29550076 PMC5993989

[B101] Wherrett D. K. Bundy B. Becker D. J. DiMeglio L. A. Gitelman S. E. Goland R. (2011). Antigen-based Therapy with Glutamic Acid Decarboxylase (GAD) Vaccine in Patients with Recent-Onset Type 1 Diabetes: a Randomised Double-Blind Trial. Lancet 378, 319–327. 10.1016/s0140-6736(11)60895-7 PubMed Abstract | 10.1016/s0140-6736(11)60895-7 | Google Scholar 21714999 PMC3580128

[B102] Wu Z. Parry M. Hou X.-Y. Liu M.-H. Wang H. Cain R. (2020). Gene Therapy Conversion of Striatal Astrocytes into GABAergic Neurons in Mouse Models of Huntington's Disease. Nat. Commun. 11, 1105. 10.1038/s41467-020-14855-3 PubMed Abstract | 10.1038/s41467-020-14855-3 | Google Scholar 32107381 PMC7046613

[B103] Xiao X. Guo P. Shiota C. Zhang T. Coudriet G. M. Fischbach S. (2018). Endogenous Reprogramming of Alpha Cells into Beta Cells, Induced by Viral Gene Therapy, Reverses Autoimmune Diabetes. Cell Stem Cell 22, 78–90. 10.1016/j.stem.2017.11.020 PubMed Abstract | 10.1016/j.stem.2017.11.020 | Google Scholar 29304344 PMC5757249

[B104] Xue Y. Ouyang K. Huang J. Zhou Y. Ouyang H. Li H. (2013). Direct Conversion of Fibroblasts to Neurons by Reprogramming PTB-Regulated microRNA Circuits. Cell 152, 82–96. 10.1016/j.cell.2012.11.045 PubMed Abstract | 10.1016/j.cell.2012.11.045 | Google Scholar 23313552 PMC3552026

[B105] Yagi M. Ji F. Charlton J. Cristea S. Messemer K. Horwitz N. (2021). Dissecting Dual Roles of MyoD during Lineage Conversion to Mature Myocytes and Myogenic Stem Cells. Genes. Dev. 35, 1209–1228. 10.1101/gad.348678.121 PubMed Abstract | 10.1101/gad.348678.121 | Google Scholar 34413137 PMC8415322

[B106] Yamakawa H. Ieda M. (2021). Cardiac Regeneration by Direct Reprogramming in This Decade and beyond. Inflamm. Regen. 41, 20. 10.1186/s41232-021-00168-5 PubMed Abstract | 10.1186/s41232-021-00168-5 | Google Scholar 34193320 PMC8247073

[B107] Young C. S. Hicks M. R. Ermolova N. V. Nakano H. Jan M. Younesi S. (2016). A Single CRISPR-Cas9 Deletion Strategy that Targets the Majority of DMD Patients Restores Dystrophin Function in hiPSC-Derived Muscle Cells. Cell. Stem Cell. 18, 533–540. 10.1016/j.stem.2016.01.021 PubMed Abstract | 10.1016/j.stem.2016.01.021 | Google Scholar 26877224 PMC4826286

[B108] Yu C. Liu K. Tang S. Ding S. (2014). Chemical Approaches to Cell Reprogramming. Curr. Opin. Genet. Dev. 28, 50–56. 10.1016/j.gde.2014.09.006 PubMed Abstract | 10.1016/j.gde.2014.09.006 | Google Scholar 25461450 PMC4747244

[B109] Zaret K. S. Grompe M. (2008). Generation and Regeneration of Cells of the Liver and Pancreas. Science 322, 1490–1494. 10.1126/science.1161431 PubMed Abstract | 10.1126/science.1161431 | Google Scholar 19056973 PMC2641009

[B110] Zhang K. Hocker J. D. Miller M. Hou X. Chiou J. Poirion O. B. (2021). A Single-Cell Atlas of Chromatin Accessibility in the Human Genome. Cell. 184, 5985–6001. e5919. 10.1016/j.cell.2021.10.024 PubMed Abstract | 10.1016/j.cell.2021.10.024 | Google Scholar 34774128 PMC8664161

[B111] Zhang L. Yin J.-C. Yeh H. Ma N.-X. Lee G. Chen X. A. (2015). Small Molecules Efficiently Reprogram Human Astroglial Cells into Functional Neurons. Cell. Stem Cell. 17, 735–747. 10.1016/j.stem.2015.09.012 PubMed Abstract | 10.1016/j.stem.2015.09.012 | Google Scholar 26481520 PMC4675726

[B112] Zhang W. Chronis C. Chen X. Zhang H. Spalinskas R. Pardo M. (2019). The BAF and PRC2 Complex Subunits Dpf2 and Eed Antagonistically Converge on Tbx3 to Control ESC Differentiation. Cell. Stem Cell. 24, 138–152. e138. 10.1016/j.stem.2018.12.001 PubMed Abstract | 10.1016/j.stem.2018.12.001 | Google Scholar 30609396 PMC6486830

[B113] Zhang X. Cruz F. D. Terry M. Remotti F. Matushansky I. (2013). Terminal Differentiation and Loss of Tumorigenicity of Human Cancers via Pluripotency-Based Reprogramming. Oncogene 32, 2249–2260. 10.1038/onc.2012.237 PubMed Abstract | 10.1038/onc.2012.237 | Google Scholar 22777357 PMC3470785

[B114] Zhang Y. Cao N. Huang Y. Spencer C. I. Fu J.-d. Yu C. (2016). Expandable Cardiovascular Progenitor Cells Reprogrammed from Fibroblasts. Cell. Stem Cell. 18, 368–381. 10.1016/j.stem.2016.02.001 PubMed Abstract | 10.1016/j.stem.2016.02.001 | Google Scholar 26942852 PMC5826660

[B115] Zhao Y. Zhao T. Guan J. Zhang X. Fu Y. Ye J. (2015). A XEN-like State Bridges Somatic Cells to Pluripotency during Chemical Reprogramming. Cell. 163, 1678–1691. 10.1016/j.cell.2015.11.017 PubMed Abstract | 10.1016/j.cell.2015.11.017 | Google Scholar 26686652

[B116] Zhou H. Su J. Hu X. Zhou C. Li H. Chen Z. (2020). Glia-to-Neuron Conversion by CRISPR-CasRx Alleviates Symptoms of Neurological Disease in Mice. Cell. 181, 590–603. 10.1016/j.cell.2020.03.024 PubMed Abstract | 10.1016/j.cell.2020.03.024 | Google Scholar 32272060

[B117] Zhou L. Ge J. Wang M. Chen M. Cheng W. Ji W. (2021). Injectable Muscle-Adhesive Antioxidant Conductive Photothermal Bioactive Nanomatrix for Efficiently Promoting Full-Thickness Skeletal Muscle Regeneration. Bioact. Mater. 6, 1605–1617. 10.1016/j.bioactmat.2020.11.005 PubMed Abstract | 10.1016/j.bioactmat.2020.11.005 | Google Scholar 33294737 PMC7691551

[B118] Zhou Q. Brown J. Kanarek A. Rajagopal J. Melton D. A. (2008). *In Vivo* reprogramming of Adult Pancreatic Exocrine Cells to β-cells. Nature 455, 627–632. 10.1038/nature07314 PubMed Abstract | 10.1038/nature07314 | Google Scholar 18754011 PMC9011918

[B119] Zhou Q. Melton D. A. (2018). Pancreas Regeneration. Nature 557, 351–358. 10.1038/s41586-018-0088-0 PubMed Abstract | 10.1038/s41586-018-0088-0 | Google Scholar 29769672 PMC6168194

